# Low- versus high-concentration iodine contrast media for coronary computed tomography angiography and dynamic stress computed tomography perfusion: a study protocol for a prospective randomized trial

**DOI:** 10.1186/s44348-026-00082-9

**Published:** 2026-07-24

**Authors:** Sung-Jin Cha, Pil-Hyun Jeon, Dong-Hee Kho, Hyosung Cho, Sung Min Ko

**Affiliations:** 1 Department of Radiology, Wonju Severance Christian Hospital, Yonsei University Wonju College of Medicine, Wonju, Republic of Korea; 2 Department of Radiation Convergence Engineering, Yonsei University, Wonju, Republic of Korea; 3 Department of Radiological Science, Dawon University, Jecheon, Republic of Korea

**Keywords:** Coronary computed tomography angiography, Myocardial perfusion imaging, Contrast media, Equivalence trial, Myocardial blood flow, Clinical Trial Protocol

## Abstract

**Background:**

This prospective randomized study protocol is designed to determine whether low-concentration iodine contrast media can maintain coronary enhancement on coronary computed tomography angiography (CCTA) compared with high-concentration iodine contrast media under standardized acquisition conditions. Dynamic stress computed tomography perfusion (CTP)-derived myocardial blood flow (MBF) will be assessed as a key secondary quantitative endpoint. Exploratory diagnostic performance analyses using invasive coronary angiography (ICA) and fractional flow reserve (FFR) will be performed only in clinically indicated invasive testing subgroups.

**Methods:**

The trial plans to enroll 258 adults (age ≥ 40 years) with known or suspected coronary artery disease referred for clinically indicated cardiac computed tomography, including CCTA with planned dynamic stress CTP. Participants will be randomized 1:1 to receive low-concentration iodine contrast media (270 mg I/mL) or high-concentration iodine contrast media (350 mg I/mL). The primary endpoint is quantitative coronary enhancement on CCTA. Dynamic stress CTP-derived MBF will be evaluated as a key secondary endpoint focused on quantitative comparability. ICA/FFR-based diagnostic performance analyses will be considered prespecified exploratory analyses in participants who undergo invasive testing as part of routine clinical care. The primary endpoint will be analyzed using linear mixed-effects models with a prespecified noninferiority margin, and MBF will be analyzed using mixed-effects models without claiming formal equivalence or noninferiority.

**Discussion:**

This study protocol is expected to clarify whether low-concentration iodine contrast media can maintain CCTA coronary enhancement and support quantitative dynamic stress CTP assessment while reducing iodine exposure. Because ICA and FFR are clinically driven rather than protocol mandated, diagnostic performance analyses will be interpreted cautiously and regarded as exploratory.

**Trial registration:**

CRIS identifier: KCT0011418. Registered on January 7, 2026.

## Background

Coronary computed tomography angiography (CCTA) has become a first-line noninvasive test for evaluating coronary artery disease (CAD), providing high diagnostic accuracy for detecting luminal stenosis and characterizing atherosclerotic plaque [1–3]. Nevertheless, CCTA has important inherent limitations. It offers a predominantly anatomic assessment of the coronary lumen and does not directly quantify the hemodynamic significance of individual lesions; consequently, the correlation between anatomic stenosis severity and functionally significant ischemia is at best modest [4]. Image quality and diagnostic performance may also be substantially degraded in patients with heavy coronary calcification or prior stents, where blooming artifacts can overestimate stenosis and increase false-positive rates [5, 6].

Dynamic stress computed tomography perfusion (CTP) is a functional imaging technique that quantifies the hemodynamic significance of coronary stenosis by measuring myocardial blood flow (MBF) under pharmacologic stress. When combined with CCTA in a single examination, dynamic stress CTP adds functional data to anatomic information, enabling a more comprehensive characterization of CAD than either modality alone and potentially improving diagnostic confidence and clinical decision-making [7–9]. This comprehensive strategy poses a dilemma in contrast use. Although high-concentration iodinated media have been favored to maximize coronary opacification, distal vessel visualization, side-branch conspicuity, and diagnostic confidence, they also increase the total iodine load, an important modifiable factor in patients at risk for contrast-associated acute kidney injury [10–12]. This issue is particularly relevant in patients with renal impairment, diabetes, or cardiovascular comorbidities.

Recent advances in computed tomography (CT) hardware and software have challenged the traditional linkage between iodine concentration and image quality. In particular, low-tube-voltage techniques augment iodine attenuation and may allow diagnostically adequate enhancement with lower iodine concentration while reducing radiation exposure [13–16]. Previous studies have also demonstrated the feasibility of low-concentration or personalized iodine contrast protocols for CCTA [17–19]. However, the ability of a low-concentration iodine protocol to maintain distal coronary visualization, side-branch conspicuity, overall CCTA diagnostic confidence, and dynamic stress CTP interpretability remains to be determined and is a key motivation for the present randomized protocol. Although dynamic stress CTP-derived MBF is primarily a physiology-based quantitative parameter, contrast iodine concentration may influence attenuation amplitude, image noise, arterial input function quality, time-attenuation curves, and post-processing robustness. Therefore, empirical evaluation of MBF comparability between low- and high-concentration iodine protocols is scientifically justified.

For a contrast media optimization protocol intended for clinical translation, technical image quality assessment alone is not sufficient. It is also important to predefine how downstream invasive coronary angiography (ICA) and fractional flow reserve (FFR) data, when obtained as part of routine care, will be analyzed. In the present study, such invasive reference standard analyses are prespecified as exploratory and hypothesis-generating because invasive testing is clinically indicated rather than mandated by the protocol.

## Methods

### Ethics statement

The protocol has been approved by the Institutional Review Board of Wonju Severance Christian Hospital (No. CR122087). The trial will be conducted in accordance with the Declaration of Helsinki and the International Council for Harmonization Good Clinical Practice guidelines. Written informed consent will be obtained from all participants before any study procedures. The trial was registered at the Clinical Research Information Service (CRIS identifier: KCT0011418).

### Trial design

**Fig. 1:**
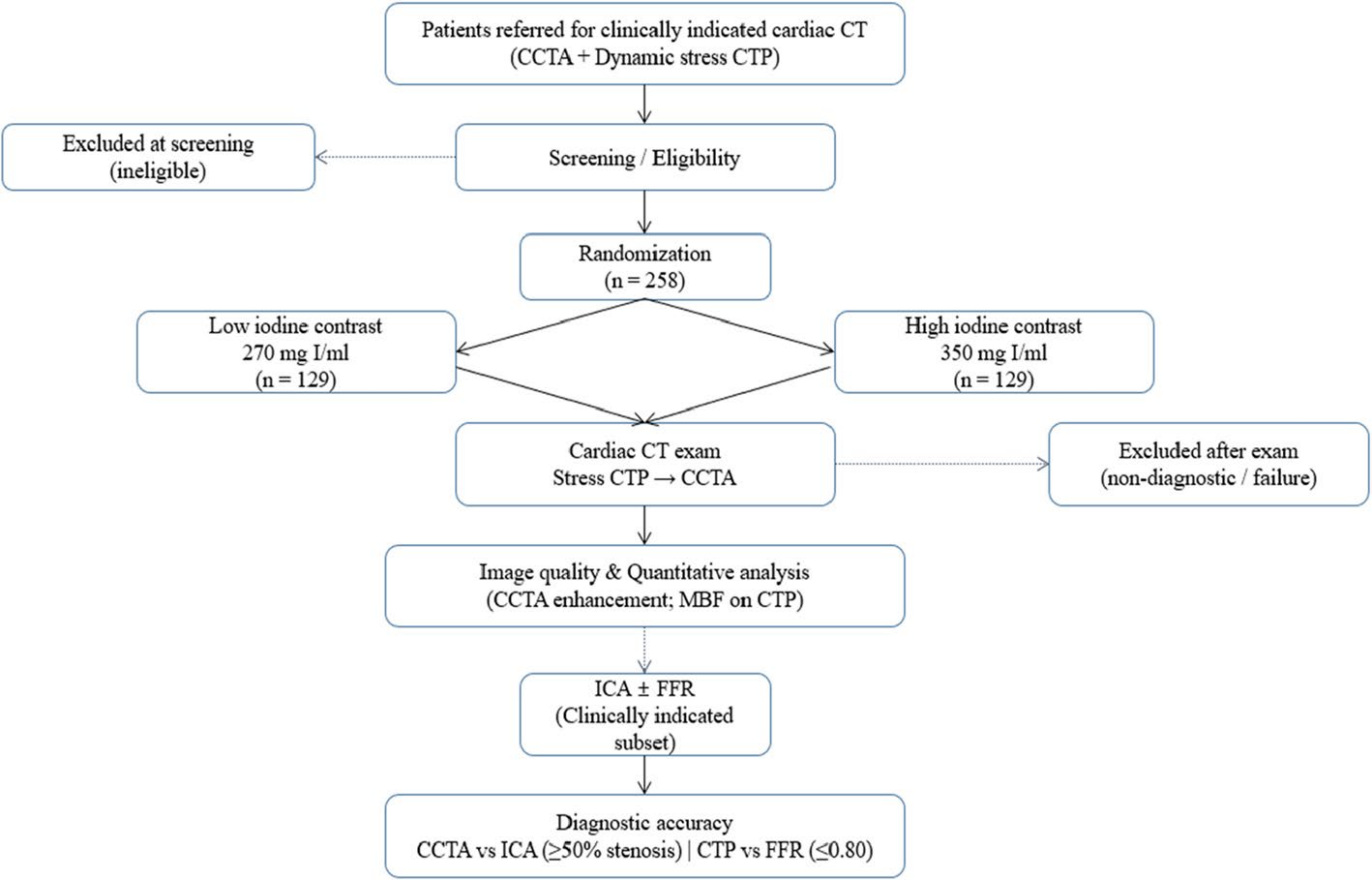
Study flow diagram and design. CCTA, coronary computed tomography angiography; CT, computed tomography; CTP, computed tomography perfusion; FFR, fractional flow reserve; ICA, invasive coronary angiography; MBF, myocardial blood flow

This study protocol describes a prospective, single-institution, randomized, participant-, investigator-, and reader-blinded noninferiority trial (Fig. 1). The primary objective is to evaluate whether a low-concentration iodine contrast protocol is noninferior to a high-concentration iodine protocol for quantitative coronary enhancement on CCTA. Dynamic stress CTP-derived MBF will be evaluated as a key secondary quantitative endpoint focused on comparability. ICA/FFR-based diagnostic performance analyses will be considered prespecified exploratory analyses in clinically indicated invasive testing subgroups.

### Population and setting

The trial plans to enroll 258 adults with known or suspected CAD referred for clinically indicated cardiac CT, including CCTA with planned dynamic stress CTP. Eligible participants will be ≥ 40 years of age, will have electrocardiography (ECG)-gated imaging feasibility, and will have adequate renal function (e.g., estimated glomerular filtration rate [eGFR] ≥ 45 mL/min/1.73 m^2^). Exclusion criteria will include suspected acute myocardial infarction or unstable angina; complex congenital heart disease; significant renal dysfunction or acute kidney injury (e.g., serum creatinine ≥ 1.5 mg/dL or eGFR < 45 mL/min/1.73 m^2^); history of coronary artery bypass grafting (percutaneous coronary intervention/stents permitted and recorded); pregnancy or lactation; history of severe hypersensitivity to iodinated contrast media or vasodilator stress agents; contraindications to nitroglycerin; contraindications to adenosine triphosphate; and uncontrolled arrhythmia precluding diagnostic acquisition. Eligible participants will be recruited from the routine clinical cardiac CT population at our institution during the planned study period.

### Sample size

The sample size is based on the primary noninferiority endpoint for CCTA enhancement. Based on prior data indicating approximately a 10.77% reduction in ascending aortic attenuation with low- versus high-concentration contrast, the noninferiority margin is set at 11% (approximately 50 Hounsfield units [HU]) [14, 15]. Assuming a standard deviation (SD) of 119 HU, one-sided α = 0.025 and 90% power, 117 participants per group are required. Allowing for 10% attrition, the target enrollment is 129 participants per group (total, n = 258). Secondary MBF and exploratory diagnostic performance analyses will be interpreted in light of the available analyzable data and the number of participants undergoing clinically indicated invasive testing.

A formal pilot study was not performed, which may limit the precision of feasibility estimates before completion of the trial. However, the protocol was developed based on prior low-iodine CCTA studies and institutional experience with integrated CCTA and dynamic stress CTP, and recruitment feasibility will be monitored throughout the study period.

### Randomization, allocation concealment, and blinding

Participants will be allocated 1:1 to the low- or high-concentration iodine contrast group using a computer-generated allocation protocol prepared by the Department of Preventive Medicine. The allocation sequence will be concealed from the principal investigator, research personnel involved in recruitment and endpoint assessment, participants, image readers, and outcome assessors. The principal investigator and research investigators will not know which contrast concentration is assigned at the time of patient enrollment, image analysis, or endpoint assessment. CT technologists involved in contrast preparation and injection may be aware of the assigned contrast media because the preparations differ; however, they will not participate in patient selection, randomization, image interpretation, endpoint assessment, statistical analysis, or clinical decision-making. After acquisition, CT images will be uploaded to PACS without visible contrast-group identifiers. The principal investigator, research investigators, image readers, and outcome assessors will analyze the anonymized imaging datasets in a blinded arrangement, and the final analytic dataset will be linked to the randomized allocation list only after blinded image analysis is complete. Participants will also not be informed of their assigned contrast concentration. Therefore, the study is described as participant-, investigator-, and reader-blinded, with transparent acknowledgement of technologist unblinding during contrast preparation.

### Contrast media and injection protocols

Participants will receive either high-concentration iodine contrast media (iobitridol 350 mg I/mL; Xenetix 350, Guerbet) or low-concentration iodine contrast media (iohexol 270 mg I/mL; Iobrix 270, Taejoon Pharmaceutical). Dynamic stress CTP will use a fixed volume of 40 mL injected at 5 mL/sec followed by a 30 mL saline chaser in both groups. Therefore, under otherwise identical perfusion-acquisition conditions, the dynamic stress CTP analysis specifically tests whether iodine concentration affects quantitative MBF measurement and interpretability.

CCTA will use a triphasic protocol at 4.5 mL/sec: 0.9 mL/kg of the assigned contrast media, followed by 45 mL of a 70:30 saline-to-contrast mixture (70% saline and 30% contrast medium), and a final 30 mL saline chaser. Two intravenous lines will be secured for contrast media injection and adenosine triphosphate infusion. All injections will be performed with a dual-head power injector (Dual Shot GX7, Nemoto Kyorindo). Weight-based dosing and fixed scan timing are intended to standardize iodine delivery conditions across participants.

### Patient preparation and stress protocol

Participants will be instructed to abstain from caffeine for 24 h prior to imaging and to withhold theophylline on the day of the examination. In the absence of contraindications, sublingual nitroglycerin will be administered immediately before CCTA to promote coronary vasodilation. For heart rate control, if baseline heart rate is ≥ 60/min, oral ivabradine (5 mg or 7.5 mg) will be administered per protocol. Heart rate will be measured every 10 min for 40 min; scanning will typically commence at approximately 60 min once adequate rate reduction is achieved. Dynamic stress CTP will be performed during intravenous adenosine triphosphate infusion at 140 μg/kg/min for 3 min.

### CT acquisition protocol

**Fig. 2:**
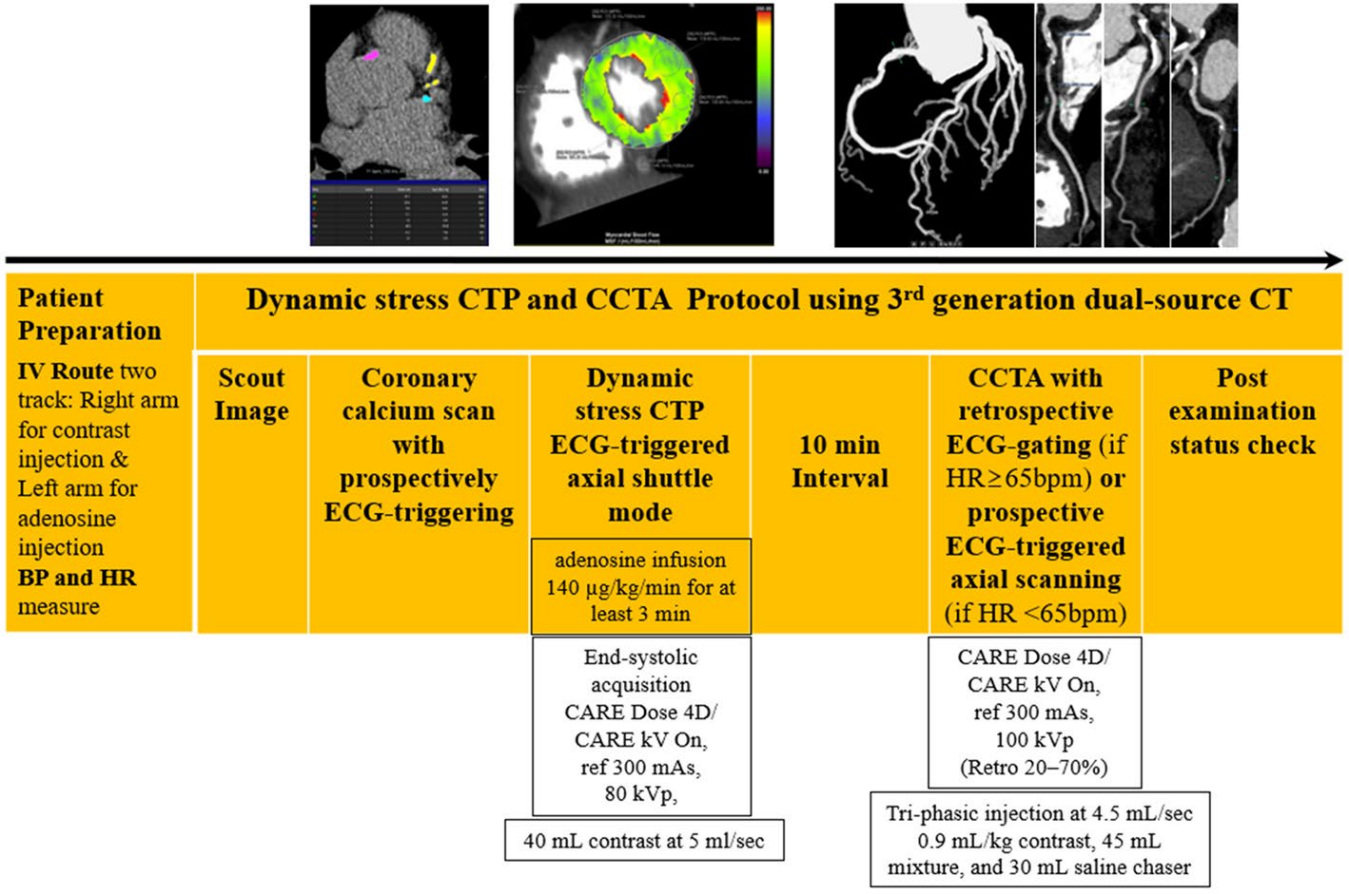
Integrated anatomic and functional assessment of coronary artery disease using coronary computed tomography angiography (CCTA) and dynamic stress computed tomography perfusion (CTP). After patient preparation and monitoring, noncontrast prospective electrocardiography-guided axial computed tomography (CT) is obtained for calcium scoring. Dynamic stress CTP is acquired in electrocardiography (ECG)-triggered axial shuttle mode during intravenous adenosine triphosphate infusion. CCTA is subsequently performed using prospective ECG-triggered or retrospective ECG-gated scanning based on heart rate. BP, blood pressure; CM, contrast media; HR, heart rate; IV, intravenous

All examinations will be performed on a third-generation dual-source CT scanner (SOMATOM Force, Siemens Healthineers) and will comprise coronary calcium scoring, dynamic stress CTP, and CCTA (Fig. 2). For dynamic stress CTP, acquisition will begin 4 s after contrast initiation and proceed in shuttle mode (z-axis coverage 105 mm) over a fixed 32-s interval, yielding approximately 10 to 15 time frames depending on heart rate. Images will be acquired in end-systole using collimation of 96× 0.6 mm and rotation time of 250 ms. Tube voltage will be selected using automatic tube-voltage selection (CARE kV, Siemens Healthineers), and tube current will be modulated using CARE Dose4D (Siemens Healthineers), according to the same institutional cardiac CT protocol in both contrast groups. Physiologic data, including baseline heart rate, peak heart rate during CTP, and heart rate increment, will be recorded prospectively.

Following completion of dynamic stress CTP, CCTA will be acquired after a standardized inter-exam interval of approximately 10 min. Immediately before CCTA, 0.6 mg sublingual nitroglycerin will be administered. For CCTA timing, bolus tracking will be used with a region of interest placed in the ascending aorta. Scanning will be triggered when attenuation reaches the predefined institutional threshold of 100 HU, followed by a 5-s post-trigger delay. The same bolus-tracking method, trigger threshold, and delay will be applied to both contrast groups. Prospective ECG-triggered axial scanning will be used for heart rate < 65/min, whereas retrospective ECG-gated acquisition with ECG-based tube-current modulation will be used for heart rate ≥ 65/min. The selected tube voltage, CT dose index volume (CTDI_vol_), and dose length product (DLP) will be recorded for each examination.

### CT reconstruction and post-processing

Dynamic stress CTP datasets will be reconstructed in the axial plane (slice thickness 3 mm, 2 mm overlap) using a medium-sharp quantitative kernel (Qr40, Siemens Healthineers) to preserve linearity of iodine attenuation. Post-processing will be performed on a workstation (Syngo.via VB10, Siemens Healthineers) using commercial perfusion software. MBF (milliliters per 100 mL/min) will be derived by parametric deconvolution based on a two-compartment tracer-kinetic model applied to time-attenuation curves. The arterial input function will be placed in the ascending aorta or left ventricular cavity using standardized regions of interest (ROIs). The left ventricle will be segmented according to the American Heart Association (AHA) 17-segment model and polar maps generated for territorial and segmental analyses.

CCTA datasets will be reconstructed with a medium-soft vascular kernel (Bv40, Siemens Healthineers) and iterative reconstruction (ADMIRE strength 3, Siemens Healthineers). Images will be transferred to an offline workstation for post-processing, and optimal phases (mid-diastolic and/or end-systolic) will be selected for evaluation. Dynamic stress CTP and CCTA acquisition parameters, including tube-voltage strategy, ECG-gating approach, reconstruction kernel, slice thickness, and iterative reconstruction settings, will be applied identically in both contrast groups to support reproducibility and minimize confounding by scan parameters.

### Radiation dose assessment

For each examination, CTDI_vol_ and DLP will be recorded for each scan component (calcium scoring, dynamic stress CTP, and CCTA). Effective dose will be estimated as DLP × 0.014 mSv/mGy·cm, and total study dose will be calculated as the sum across components.

### Image analysis

**Fig. 3:**
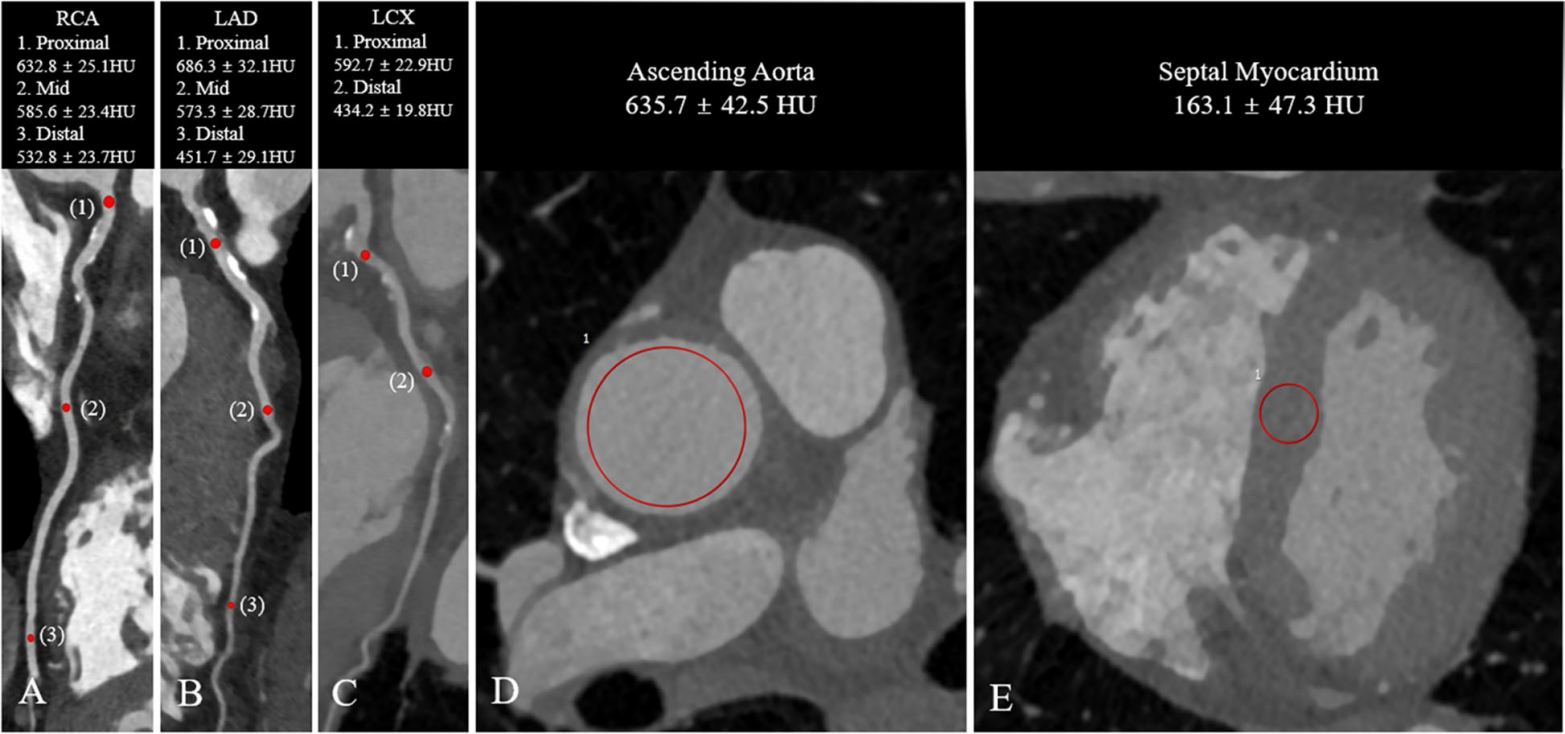
Quantitative assessment of coronary computed tomography angiography (CCTA) image quality. **A**–**C** Curved multiplanar reformations of the right coronary artery (RCA), left anterior descending artery (LAD), and left circumflex artery (LCX), showing regions of interest (ROIs) placed in the proximal and distal coronary segments for attenuation measurement. **D** Ascending aortic ROI used for image noise measurement. **E** Septal myocardial ROI used for myocardial attenuation measurement. HU, Hounsfield units

Quantitative CCTA image quality will be evaluated using ROIs in proximal and distal segments of each major coronary artery (left anterior descending coronary artery, left circumflex artery, right coronary artery) to obtain mean attenuation (HU) (Fig. 3A–C). Image noise will be defined as the SD of attenuation in a homogeneous ascending aortic ROI (Fig. 3D). Signal to noise ratio (SNR) and contrast to noise ratio (CNR) will be calculated as SNR = HU_coronary_/SD_aorta_ and CNR = (HU_coronary_ – HU_myocardium_)/SD_aorta_, where HU_myocardium_ is measured from a mid-ventricular septal ROI (Fig. 3E). Measurements will be performed on the optimal phase and averaged for per-vessel and per-patient analyses.

**Fig. 4:**
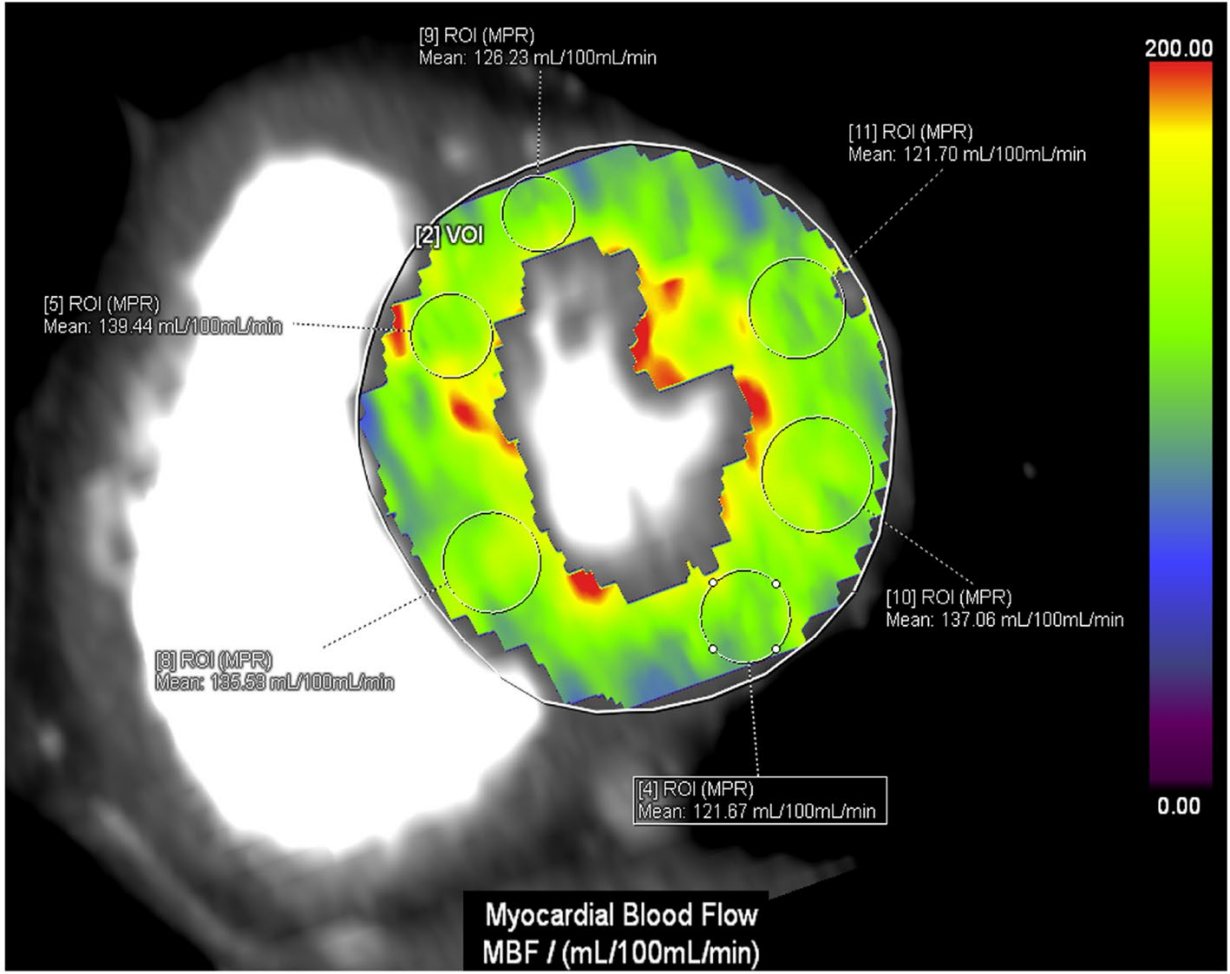
Quantitative assessment of myocardial blood flow (MBF) using dynamic stress computed tomography perfusion (CTP). Short-axis MBF map obtained from dynamic stress CTP, with segmental regions of interest (ROIs) placed according to the American Heart Association 17-segment model

Dynamic stress CTP-derived MBF will be quantified on voxel-wise MBF maps. For segmental analysis, ROIs will be drawn on short-axis MBF maps using the AHA 17-segment model, positioned ≥ 2 mm from endocardial and epicardial borders (Fig. 4). To avoid circularity, MBF thresholds will not be used to define the main MBF analysis set. The main analysis will include all analyzable myocardial segments, with sensitivity analyses in visually nonischemic segments and, when available, territories not supplied by vessels with significant anatomic stenosis or FFR-defined ischemia.

### Qualitative image quality assessment

All CCTA and dynamic stress CTP datasets will be independently reviewed by two board-certified cardiovascular radiologists with more than 10 years of experience who are blinded to clinical information and contrast-group assignments. Coronary image quality will be assessed on axial, multiplanar, and curved planar reformation images using a 5-point scale: 5, excellent visualization without relevant artifact; 4, good visualization with minor artifact; 3, adequate visualization with moderate artifact but diagnostic; 2, poor visualization with severe artifact and limited diagnostic confidence; and 1, nondiagnostic image quality. Volume-rendered images will be reviewed as supplementary overview images for global coronary visualization, distal vessel/side-branch conspicuity, and overall diagnostic confidence, but will not be used alone for stenosis assessment. Dynamic stress CTP interpretability will be graded using an analogous 5-point scale. Readers will also record diagnostic acceptability (acceptable vs. nonacceptable) and diagnostic confidence (high, medium, or low). Discrepancies will be resolved by consensus after independent review.

### Exploratory diagnostic performance analyses: ICA and FFR

Exploratory diagnostic performance analyses will be performed in participants who undergo ICA and/or FFR as part of routine clinical care. The decision to perform CCTA with dynamic stress CTP, as well as subsequent ICA with or without FFR, will be made by the treating cardiologists according to routine clinical care and will not be dictated by study participation. Accordingly, the primary purpose of this trial is to compare contrast media protocols for CCTA enhancement and quantitative dynamic stress CTP assessment, rather than to establish diagnostic validity of CCTA or CTP.

ICA will be interpreted by experienced interventional cardiologists. Anatomic stenosis severity will be recorded per vessel using standard quantitative or visual assessment; ≥ 50% luminal narrowing will define significant stenosis for exploratory CCTA diagnostic performance analyses. For physiology, when FFR is measured, FFR ≤ 0.80 will define functionally significant ischemia in the interrogated vessel. For CCTA diagnostic performance analyses, CCTA-derived stenosis categories will be compared with ICA on a per-vessel and per-patient basis. For dynamic stress CTP diagnostic performance analyses, CTP ischemia (territorial/segmental defects or low MBF) will be compared with vessel-level FFR where available, with mapping of myocardial territories to corresponding coronary vessels using standardized AHA territorial assignments and clinical correlation. Because invasive testing is clinically indicated rather than protocol mandated for all participants, these analyses are subject to potential verification bias and will be interpreted cautiously as prespecified exploratory and hypothesis-generating analyses.

### Statistical methods

All statistical analyses will be performed using SAS ver. 9.4 (SAS Institute Inc). Baseline characteristics will be summarized by group. Standardized mean differences (SMDs) will be used to assess baseline balance; variables with absolute SMD ≥ 0.25 will be considered imbalanced and included as covariates in sensitivity analyses.

Quantitative endpoints with repeated measures (HU, SNR, CNR, and MBF) will be analyzed using linear mixed-effects models with random intercepts for patients to account for intraindividual correlation across vessels or myocardial segments. Fixed effects will include contrast group, measurement location, and group × location interaction where appropriate. For the primary CCTA enhancement endpoint, noninferiority will be assessed using the estimated between-group difference (low minus high) and its two-sided 95% confidence interval. Noninferiority will be concluded if the lower bound of the 95% confidence interval for ΔHU is greater than –50 HU and if diagnostic adequacy criteria are met.

For the key secondary MBF endpoint, between-group differences will be estimated using a linear mixed-effects model. Because no clinically justified noninferiority or equivalence margin is prespecified for MBF, the analysis will be interpreted as an assessment of quantitative comparability rather than formal equivalence or noninferiority. A nonsignificant between-group difference will not be interpreted as proof of equivalence.

Qualitative image quality endpoints (Likert scores) will be compared using Mann–Whitney U-tests, with proportional-odds models as sensitivity analyses. Interobserver agreement will be assessed using weighted kappa for ordinal scales and Cohen κ for binary acceptability.

Exploratory diagnostic performance analyses will be conducted in the subset undergoing ICA and/or FFR. For CCTA using ICA as the reference standard, sensitivity, specificity, positive predictive value, and negative predictive value will be calculated for the detection of ≥ 50% coronary artery stenosis. Overall diagnostic performance will be quantified using receiver operating characteristic (ROC) curve analysis and the area under the ROC curve (AUC). For dynamic stress CTP versus FFR, similar metrics will be calculated for detecting FFR-defined ischemia (≤ 0.80). Because multiple vessels may be contributed by a single participant, analyses will account for within-patient clustering using methods such as generalized estimating equations for binary endpoints and cluster-robust variance estimators for AUC and confidence intervals. Prespecified sensitivity analyses will evaluate the impact of verification bias, as feasible given sample size. Missing data will be described and handled using appropriate methods for the endpoint, including mixed models under missing-at-random assumptions with sensitivity analyses if missingness is substantial.

### Data management and safety monitoring

A centralized data-coordinating center will oversee data capture and quality control. Study data will be entered into a secure electronic system. Monitoring will include periodic audits of completeness, protocol adherence, and adverse-event reporting. Endpoints are imaging-based and do not mandate repeat imaging. Clinical management decisions, including invasive testing, will be determined by treating physicians according to standard care. No interim efficacy analysis is planned.

## Discussion

This prospective randomized study protocol addresses a clinically important question in contemporary cardiac CT: whether reduction of iodine concentration in contrast media can maintain CCTA coronary enhancement under standardized acquisition conditions while supporting quantitative dynamic stress CTP assessment. The revised endpoint hierarchy intentionally focuses the trial on contrast media optimization rather than on establishing the intrinsic diagnostic validity of CCTA or dynamic stress CTP.

CCTA is widely used as a first-line noninvasive test for suspected CAD because of its high sensitivity and negative predictive value [16, 20]. However, false-positive findings remain a concern, particularly in patients with heavy coronary calcification, diffuse atherosclerosis, or prior percutaneous coronary intervention [5, 6]. In these settings, distal vessel visualization, side-branch conspicuity, and diagnostic confidence remain important image quality considerations when iodine concentration is reduced.

Dynamic stress CTP provides complementary functional information by enabling quantitative assessment of MBF and ischemia, thereby addressing one of the key limitations of anatomic imaging [7–9]. Although MBF is fundamentally a physiology-derived and model-based parameter, iodine concentration may affect time-attenuation curve amplitude, image noise, arterial input function quality, and segmentation or post-processing robustness. Therefore, assessing MBF comparability under otherwise identical acquisition and injection conditions is justified.

The integration of CCTA and dynamic stress CTP into a single examination offers a comprehensive evaluation of both coronary anatomy and myocardial perfusion. However, this approach increases total iodine exposure, which remains clinically relevant in older patients and in those with chronic kidney disease, diabetes, or cardiovascular comorbidities [10–12]. If low-concentration iodine contrast media maintain coronary enhancement and support quantitative MBF assessment, the protocol may help reduce iodine exposure while preserving the technical basis for integrated anatomic-functional cardiac CT evaluation.

Several limitations warrant consideration. First, this is a single-center study, and generalizability may depend on scanner technology, acquisition protocol, reconstruction settings, and local expertise. Second, a formal pilot study was not performed, which may limit the precision of feasibility estimates before completion of enrollment. Third, ICA and FFR are performed based on clinical indication rather than mandated by the protocol, introducing potential verification bias. For this reason, ICA/FFR-based diagnostic performance analyses are prespecified as exploratory and hypothesis-generating. Finally, the study focuses on imaging-based endpoints and does not evaluate clinical outcomes; therefore, the results will inform image quality and quantitative perfusion assessment validity rather than outcome equivalence.

## Conclusions

This study protocol describes a prospective, randomized, participant-, investigator-, and reader-blinded noninferiority trial designed to evaluate whether low-concentration iodine contrast media can maintain CCTA coronary enhancement under standardized cardiac CT acquisition conditions. Dynamic stress CTP-derived MBF will be assessed as a key secondary quantitative endpoint, and ICA/FFR-based diagnostic performance analyses will be interpreted cautiously as prespecified exploratory analyses in clinically indicated invasive testing subgroups. The study is expected to provide evidence supporting a more patient-centered and resource-conscious approach to contrast media optimization in integrated CCTA and dynamic stress CTP.

## Data Availability

No datasets were generated or analysed during the current study.

## Abbreviations

AHA: American Heart Association
AUC: Area under the ROC curve
CAD: Coronary artery disease
CCTA: Coronary computed tomography angiography
CNR: Contrast to noise ratio
CT: Computed tomography
CTDI_vol_: Computed tomography dose index volume
CTP: Computed tomography perfusion
DLP: Dose length product
ECG: Electrocardiography
eGFR: Estimated glomerular filtration rate
FFR: Fractional flow reserve
HU: Hounsfield units
ICA: Invasive coronary angiography
MBF: Myocardial blood flow
ROC: Receiver operating characteristic
ROI: Region of interest
SD: Standard deviation
SMD: Standardized mean difference
SNR: Signal to noise ratio
